# Supplementary material for: Quantitative system drift compensates for altered maternal inputs to the gap gene network of the scuttle fly Megaselia abdita
Source: eLife. 2015 Jan 5;4:e04785. doi: 10.7554/eLife.04785 (PMC4337606; doi:10.7554/eLife.04785)
Supplement: Supplementary file 1. — Maternal co-ordinate and terminal gap gene mRNA expression dataset for M. abdita. This file contains a table with numbers of embryos in our dataset for maternal co-ordinate and terminal gap gene mRNA expression in M. abdita. DOI: http://dx.doi.org/10.7554/eLife.04785.018 [file elife04785s001.docx]

**Supplementary File 1:**

**Maternal co-ordinate and terminal gap gene mRNA expression data set for *M. abdita*.**

The number of embryos used in the analysis of each indicated expression domain at each time class is shown. Time classification as defined in [1]: C11–13 correspond to cleavage cycles 11 to 13; T1–8 represent time classes subdividing C14A.

| **Time Class** | ***bcd:*** | ***cad:* abdominal domain** | ***cad:* posterior stripe** | ***tll:* posterior domain** | ***hkb:* posterior domain** |
| --- | --- | --- | --- | --- | --- |
| **C11** | 4 | - | - | - | - |
| **C12** | 9 | 3 | - | 1 | 2 |
| **C13** | 2 | 6 | - | 5 | 1 |
| **T1** | 2 | 3 | - | 4 | 3 |
| **T2** | - | 6 | - | 4 | 4 |
| **T3** | - | 5 | 1 | 8 | 7 |
| **T4** | - | 7 | 6 | 8 | - |
| **T5** | - | 2 | 2 | 3 | 5 |
| **T6** | - | 1 | 4 | - | 3 |
| **T7** | - | - | 2 | - | 1 |
| **T8** | - | - | 5 | - | 6 |
| **Total:** | 17 | 33 | 20 | 33 | 32 |

1. Wotton KR, Jiménez-Guri E, García Matheu B, Jaeger J (2014) A Staging Scheme for the Development of the Scuttle Fly *Megaselia abdita.* PLoS ONE 9: e84421.
