# Supplementary material for: Quantitative system drift compensates for altered maternal inputs to the gap gene network of the scuttle fly Megaselia abdita
Source: eLife. 2015 Jan 5;4:e04785. doi: 10.7554/eLife.04785 (PMC4337606; doi:10.7554/eLife.04785)
Supplement: Supplementary file 2. — Boundary positions/shifts and domain widths/overlaps contain tables with numerical comparisons of expression data between M. abdita and D. melanogaster. (A) Comparison of expression data between M. abdita and D. melanogaster: gap domain boundary positions. (B) Comparison of expression data between M. abdita and D. melanogaster: gap domain widths. (C) Comparison of expression data between M. abdita and D. melanogaster: gap domain boundary shifts. (D) Comparison of expression data between M. abdita and D. melanogaster: gap domain overlaps. DOI: http://dx.doi.org/10.7554/eLife.04785.019 [file elife04785s002.docx]

# Supplementary File 2: Boundary Positions/Shifts and Domain Widths/Overlaps

**Supplementary File 2A. Comparison of expression data between *M. abdita* and *D. melanogaster*: gap domain boundary positions.** This table shows boundary locations in percent A–P position (where 0% is the anterior pole) through developmental time. Black numbers represent *M. abdita* expression boundaries, grey numbers those for *D. melanogaster*. A indicates anterior, P posterior boundary of a domain. Time classification as defined in [1] for *M. abdita*, and in [2] for *D. melanogaster*: C11–13 correspond to cleavage cycles 11 to 13; T1–8 represent time classes subdividing C14A. Boundary positions correspond to starting points of approximating splines as described in [3]. Dashes indicate boundaries that are not present at a given time point. Italics indicate incompletely formed boundaries. The *D. melanogaster* gap gene data set has been published previously [3]. It is included here for comparison.

| **Domain** | ***hb:* anterior** | ***gt:***  **anterior** | | ***Kr:***  **central** | | ***kni:***  **abdominal** | | ***gt:***  **posterior** | | ***hb:***  **posterior** | |
| --- | --- | --- | --- | --- | --- | --- | --- | --- | --- | --- | --- |
|  | **P** | **A** | **P** | **A** | **P** | **A** | **P** | **A** | **P** | **A** | **P** |
| **C12** | 50.2 | 14.0 | 33.7 | 39.6 | 62.6 | 60.6 | 77.6 | 73.3 | - | 90.2 | - |
|  | 44.8 | - | - | 48.3 | 60.0 | - | - | - | - | - | - |
| **C13** | 48.5 | 15.3 | 34.5 | 40.2 | 63.5 | 58.5 | 74.5 | 72.0 | 88.7 | 88.7 | - |
|  | 45.8 | 19.3 | 39.8 | 46.7 | 59.4 | 58.3 | 72.0 | 74.0 | - | 89.5 | - |
| **T1** | 45.0 | 13.6 | 32.5 | 40.2 | 61.1 | 57.4 | 71.0 | 69.7 | 86.2 | 86.1 | - |
|  | 43.8 | 20.7 | 39.3 | 45.3 | 57.8 | 56.5 | 70.1 | 69.8 | 82.2 | 85.0 | - |
| **T2** | 44.0 | 14.7 | 34.3 | 39.9 | 60.6 | 56.3 | 71.3 | 70.6 | 86.0 | 83.9 | - |
|  | 44.4 | 20.0 | 38.2 | 44.3 | 57.3 | 57.9 | 70.1 | 69.8 | 79.4 | 81.2 | 92.2 |
| **T3** | 42.9 | 14.4 | 33.1 | 38.6 | 58.0 | 57.6 | 70.1 | 70.0 | 85.8 | 79.2 | - |
|  | 45.1 | 18.7 | 37.8 | 45.5 | 59.8 | 58.0 | 70.0 | 67.5 | 76.9 | 80.1 | 89.3 |
| **T4** | 42.2 | 16.6 | 34.9 | 38.8 | 58.0 | 55.8 | 67.8 | 68.3 | 84.0 | 78.5 | - |
|  | 47.0 | 18.2 | 37.7 | 44.0 | 57.8 | 56.9 | 67.1 | 67.0 | 77.0 | 78.9 | 88.0 |
| **T5** | 42.1 | 16.9 | 31.9 | 38.3 | 54.7 | 54.7 | 66.4 | 65.0 | 78.5 | 79.0 | - |
|  | 45.9 | 18.3 | 37.7 | 43.9 | 56.7 | 56.0 | 66.0 | 66.8 | 76.5 | 76.9 | 87.7 |
| **T6** | 41.5 | 24.0 | 33.5 | 37.7 | 51.5 | 54.2 | 65.9 | 64.4 | 74.8 | 76.1 | *86.0* |
|  | 45.8 | 18.8 | 37.3 | 43.5 | 56.4 | 54.5 | 64.8 | 65.6 | 75.1 | 75.0 | 86.6 |
| **T7** | 41.5 | 24.0 | 32.4 | 37.3 | 51.3 | 53.4 | 64.4 | 63.2 | 72.8 | 75.7 | *85.8* |
|  | 45.8 | 19.7 | 37.6 | 43.0 | 52.5 | 54.5 | 63.0 | 64.5 | 72.1 | 75.6 | 86.1 |
| **T8** | 42.2 | 25.6 | 33.2 | 37.0 | 50.7 | 53.6 | 63.5 | 62.9 | 71.0 | 72.4 | 84.1 |
|  | 45.0 | 20.5 | 37.2 | 42.7 | 52.5 | 54.7 | 63.6 | 65.3 | 72.8 | 75.5 | 85.8 |

**Supplementary File 2B. Comparison of expression data between *M. abdita* and *D. melanogaster*: gap domain widths.** This table shows the width of gap domains (in percent egg length) through developmental time. Black numbers represent *M. abdita* expression domains, grey numbers those for *D. melanogaster*. Time classification as defined in [1] for *M. abdita*, and in [2] for *D. melanogaster*: C12/13 correspond to cleavage cycles 12 and 13; T1–5 represent time classes subdividing C14A. Domain width is calculated as the difference between boundary positions (shown in Supplementary Table 2A). Dashes indicate boundaries that are not present at a given time point. Italics indicate domains that extend to the posterior pole of the embryo. ‘% Narrowing’ indicates by how much each domain contracts over time (between C13 and T8). The *D. melanogaster* gap gene data set has been published previously [3]. It is included here for comparison.

| **Domain** | ***gt:***  **anterior**  **domain** | ***Kr:***  **central**  **domain** | ***kni*:**  **abdominal**  **domain** | ***gt:***  **posterior**  **domain** | ***hb:***  **posterior**  **domain** |
| --- | --- | --- | --- | --- | --- |
| **C12** | 19.7 | 23.0 | 17.0 | - | *9.8* |
|  | - | 11.7 | - | - | *-* |
| **C13** | 19.2 | 23.3 | 16.0 | 16.7 | *11.3* |
|  | 20.5 | 12.7 | 13.7 | 26.0 | *10.5* |
| **T1** | 18.9 | 20.9 | 13.6 | 16.5 | *13.8* |
|  | 18.6 | 12.5 | 13.6 | 12.4 | *15.0* |
| **T2** | 19.6 | 20.7 | 15.0 | 15.4 | *13.9* |
|  | 18.2 | 13.0 | 12.2 | 9.6 | 11.0 |
| **T3** | 18.7 | 19.4 | 12.5 | 15.8 | *20.8* |
|  | 19.1 | 14.3 | 12.0 | 9.4 | 9.2 |
| **T4** | 18.3 | 19.2 | 12.0 | 15.7 | *21.5* |
|  | 19.5 | 13.8 | 10.2 | 10.0 | 9.1 |
| **T5** | 15.0 | 16.4 | 11.7 | 13.5 | *21.0* |
|  | 19.4 | 12.8 | 10.0 | 9.7 | 10.8 |
| **T6** | 9.5 | 13.8 | 11.7 | 10.4 | 9.9 |
|  | 18.5 | 12.9 | 10.3 | 9.5 | 11.6 |
| **T7** | 8.4 | 14.0 | 11.0 | 9.6 | 10.1 |
|  | 17.9 | 9.5 | 8.5 | 7.6 | 10.5 |
| **T8** | 7.6 | 13.7 | 9.9 | 8.1 | 11.7 |
|  | 16.7 | 9.8 | 8.9 | 7.5 | 10.3 |
| **% Narrowing** | 60% | 41% | 39% | 51% | -3% |
|  | 19% | 23% | 35% | 71% | 2% |

**Supplementary File 2C. Comparison of expression data between *M. abdita* and *D. melanogaster*: gap domain boundary shifts.** This table shows the extent of boundary shifts (in percent egg length) relative to the boundary position at C14A-T1 (light grey background), with the exception of the posterior boundary of the posterior *hb* domain, which only appears during T6 (*M. abdita*), and T2 *(D. melanogaster*). Negative numbers represent shifts towards the anterior, positive numbers shifts towards the posterior of the embryo. Black numbers represent *M. abdita* expression boundaries, grey numbers those for *D. melanogaster*. Time classification as defined in [1] for *M. abdita*, and in [2] for *D. melanogaster*: C12/13 correspond to cleavage cycles 12 and 13; T1–5 represent time classes subdividing C14A.. Dashes indicate boundaries that are not present at a given time point. ‘Total Shift’ indicates by how much each boundary has shifted overall between C12 and T8. Italics indicate shift from the posterior pole (not the initially measured boundary position at T2 or T6). The *D. melanogaster* gap gene data set has been published previously [3]. It is included here for comparison.

| **Domain** | ***hb:***  **anterior** | ***gt:***  **anterior** | | ***Kr:***  **central** | | ***kni:***  **abdominal** | | ***gt:***  **posterior** | | ***hb:***  **posterior** | |
| --- | --- | --- | --- | --- | --- | --- | --- | --- | --- | --- | --- |
|  | **P** | **A** | **P** | **A** | **P** | **A** | **P** | **A** | **P** | **A** | **P** |
| **C12** | +5.2 | +0.4 | +1.2 | -0.6 | +1.5 | +3.2 | +6.6 | +3.6 | - | +4.1 | - |
|  | +1.0 | - | - | +3.0 | +2.2 | - | - | - | - | - | - |
| **C13** | +3.5 | +1.7 | +2.0 | 0.0 | +2.4 | +1.1 | +3.5 | +2.3 | +2.5 | +2.6 | - |
|  | +2.0 | -1.4 | +0.5 | 1.4 | +1.6 | +1.8 | +1.9 | +4.2 | - | +4.5 | - |
| **T1** | 45.0 | 13.6 | 32.5 | 40.2 | 61.1 | 57.4 | 71.0 | 69.7 | 86.2 | 86.1 | - |
|  | 43.8 | 20.7 | 39.3 | 45.3 | 57.8 | 56.5 | 70.1 | 69.8 | 82.2 | 85.0 | - |
| **T2** | -1.0 | +1.1 | +1.8 | -0.3 | -0.5 | -1.1 | +0.3 | +0.9 | -0.2 | -2.2 | - |
|  | +0.6 | -0.7 | -1.1 | -1.0 | -0.5 | +1.4 | 0.0 | 0.0 | -2.8 | -3.8 | 89.7 |
| **T3** | -2.1 | +0.8 | +0.6 | -1.6 | -3.1 | +0.2 | +0.9 | +0.3 | -0.4 | -6.9 | - |
|  | +1.3 | -2.0 | -1.5 | +0.2 | +2.0 | +1.5 | -0.1 | -2.3 | -5.3 | -3.9 | -2.9 |
| **T4** | -2.8 | +3.0 | +2.4 | -1.4 | -3.1 | -1.6 | -3.2 | -1.4 | -2.2 | -7.6 | - |
|  | +3.2 | -2.5 | -1.6 | -1.3 | 0.0 | +0.4 | -3.0 | -2.8 | -5.2 | -6.1 | -4.2 |
| **T5** | -2.9 | +3.3 | -0.6 | -1.9 | -6.4 | -2.7 | -4.6 | -4.7 | -7.7 | -7.1 | - |
|  | +2.1 | -2.4 | -1.6 | -1.4 | -1.1 | -0.5 | -4.1 | -3.0 | -5.7 | -8.1 | -4.5 |
| **T6** | -3.5 | +10.4 | -1.0 | -2.5 | -9.6 | -3.2 | -5.1 | -5.3 | -11.4 | -10.0 | 86.0 |
|  | +2.0 | -1.9 | -2.0 | -1.8 | -1.4 | -2.0 | -5.3 | -4.2 | -7.1 | -10.0 | -5.6 |
| **T7** | -3.5 | +10.4 | -0.1 | -2.9 | -9.8 | -4.0 | -6.6 | -6.5 | -13.4 | -10.4 | -0.2 |
|  | +2.0 | -1.0 | -1.7 | -2.3 | -5.3 | -2.0 | -7.1 | -5.3 | -10.1 | -9.4 | -6.1 |
| **T8** | -2.8 | +12 | +0.7 | -3.2 | -10.4 | -3.8 | -7.5 | -6.8 | -15.2 | -13.7 | -1.9 |
|  | +1.2 | -0.2 | -2.1 | -2.6 | -5.3 | -1.8 | -6.5 | -4.5 | -9.4 | -9.5 | -6.4 |
| **Total** | -8.0 | +11.6 | -0.5 | -2.6 | -11.9 | -7.0 | -14.1 | -10.4 | -17.7 | -17.8 | *-15.9* |
| **Shift:** | +0.2 | +1.2 | -2.6 | -5.6 | -7.5 | -3.6 | -8.4 | -8.7 | -9.4 | -14.0 | *-16.7* |

**Supplementary File 2D. Comparison of expression data between *M. abdita* and *D. melanogaster*: gap domain overlaps.** These tables show the extent of overlap between gap domains (in percent egg length) through developmental time. Negative numbers indicate distance between non-overlapping domains. ‘Interaction’ indicates the regulatory mechanism the overlapping genes are involved in (see main text for details). Black numbers represent *M. abdita* expression domains, grey numbers those for *D. melanogaster*. Time classification as defined in [1] for *M. abdita*, and in [2] for *D. melanogaster*: C12/13 correspond to cleavage cycles 12 and 13; T1–5 represent time classes subdividing C14A. Overlaps are calculated as differences between boundary positions (shown in Supplementary Table 2A). Dashes indicate no overlap, or domain not present, at a given time point. ‘full’ means domains overlap completely. The *D. melanogaster* gap gene data set has been published previously [3]. It is included here for comparison.

| **Overlap**  **in %EL** | **ant *gt Kr*** | ***Kr***  **post *gt*** | **ant *hb kni*** | **post *hb kni*** |  | **ant *hb Kr*** | **post *hb***  **post *gt*** | **post *gt kni*** | ***Kr***  ***kni*** |  | **post *hb hkb*** | **post *hb tll*** | **post *gt hkb*** | **post *gt tll*** |
| --- | --- | --- | --- | --- | --- | --- | --- | --- | --- | --- | --- | --- | --- | --- |
| **Interaction** | alt.  cushion | alt.  cushion | alt.  cushion | alt.  cushion |  | shift | shift | shift | shift |  | terminal | terminal | terminal | terminal |
| **C12** | 4.4 | -0.8 | 1.6 | -5.8 |  | 23.7 | - | 11.5 | 13.5 |  | full | full | - | - |
|  | - | - | - | - |  | 7.0 | - | - | - |  | full | full | - | - |
| **C13** | 3.3 | 2.0 | 0.7 | -4.8 |  | 20.4 | 7.3 | 12.0 | 15.1 |  | full | full | 5.1 | 5.2 |
|  | 2.3 | -3.9 | -2.8 | -10.6 |  | 8.7 | - | 7.5 | 10.7 |  | full | full | - | - |
| **T1** | 1.0 | 1.7 | -3.2 | -5.2 |  | 15.4 | 8.7 | 10.5 | 13.7 |  | full | full | 2.8 | 4.2 |
|  | 3.1 | -2.6 | -2.7 | -6.6 |  | 9.1 | 5.9 | 8.5 | 11.1 |  | full | full | -1.8 | 1.4 |
| **T2** | 3.4 | -0.2 | -3.5 | -3.0 |  | 14.0 | 10.8 | 9.4 | 14.7 |  | full | full | 3.9 | 3.0 |
|  | 2.8 | -3.7 | -4.7 | -2.4 |  | 8.9 | 6.2 | 8.5 | 9.0 |  | 9.1 | 12.2 | -2.1 | 0.9 |
| **T3** | 2.3 | -3.3 | -7.0 | -1.9 |  | 12.6 | 13.9 | 7.4 | 9.2 |  | full | full | 2.5 | 4.1 |
|  | 0.4 | 0.0 | -5.7 | -2.0 |  | 7.4 | 4.8 | 9.9 | 10.2 |  | 7.5 | 12.7 | -3.3 | 1.9 |
| **T4** | 4.0 | -2.0 | -5.7 | -3.1 |  | 11.6 | 12.6 | 7.3 | 10.8 |  | full | full | - | 1.7 |
|  | 2.2 | -1.4 | -2.9 | -5.3 |  | 10.9 | 5.3 | 6.2 | 9.5 |  | 5.7 | 10.3 | -4.1 | 0.6 |
| **T5** | 0.1 | -4.1 | -5.5 | -5.6 |  | 11.0 | 6.6 | 7.2 | 8.0 |  | full | full | -5.0 | -4.8 |
|  | 0.7 | -2.8 | -4.5 | -4.7 |  | 7.6 | 6.3 | 5.3 | 8.8 |  | 4.7 | 8.8 | -5.4 | -1.3 |
| **T6** | 1.5 | -7.0 | -6.5 | -3.1 |  | 9.4 | 5.1 | 8.1 | 3.8 |  | 0.8 | - | -9.8 | - |
|  | -0.3 | -3.0 | -3.8 | -3.0 |  | 7.3 | 7.2 | 5.9 | 8.6 |  | 4.0 | 7.0 | -6.3 | -3.3 |
| **T7** | 1.1 | -7.2 | -5.4 | -5.3 |  | 10.8 | 2.7 | 6.2 | 4.3 |  | -1.5 | - | -13.5 | - |
|  | 0.0 | -6.3 | -3.9 | -6.3 |  | 7.0 | 2.5 | 3.7 | 5.0 |  | 2.2 | - | 11.2 | - |
| **T8** | 20 | -6.6 | -6.0 | -3.6 |  | 11.6 | 3.7 | 6.1 | 2.6 |  | -2.5 | - | -16.0 | - |
|  | -0.4 | -7.6 | -5.9 | -5.6 |  | 7.0 | 4.0 | 3.7 | 2.4 |  | 0.9 | - | -11.5 | - |

1. Wotton KR, Jiménez-Guri E, García Matheu B, Jaeger J (2014) A Staging Scheme for the Development of the Scuttle Fly *Megaselia abdita.* PLoS ONE 9: e84421.

2. Surkova S, Myasnikova E, Janssens H, Kozlov KN, Samsonova AA, Reinitz J, Samsonova M (2008). Pipeline for acquisition of quantitative data on segmentation gene expression from confocal images. Fly 2: 1–9.

3. Crombach A, Wotton KR, Cicin-Sain D, Ashyraliyev M, Jaeger J (2012). Efficient Reverse-Engineering of a Developmental Gene Regulatory Network. PLoS Comp Biol 8: e1002589.
