# Supplementary material for: Quantitative system drift compensates for altered maternal inputs to the gap gene network of the scuttle fly Megaselia abdita
Source: eLife. 2015 Jan 5;4:e04785. doi: 10.7554/eLife.04785 (PMC4337606; doi:10.7554/eLife.04785)
Supplement: Supplementary file 3. — Megaselia segmentation gene expression. DOI: http://dx.doi.org/10.7554/eLife.04785.020 [file elife04785s003.docx]

# Supplementary File 3:

# *Megaselia* Segmentation Gene Expression

This file documents the quantified wild-type mRNA expression patterns of *Megaselia abdita* maternal co-ordinate and gap genes. First, we introduce our scheme for naming/identifying boundaries. Then, we present tables with the raw data for measured boundary positions. Systematic comparisons of boundary positions, domain widths, boundary shifts, and domain overlaps between the two species can be found in Supplementary File 2, Tables A–D.

Gene abbreviations: *bicoid (bcd), caudal (cad), tailless (tll), huckebein (hkb), hunchback (hb), giant (gt), Krüppel (Kr), knirps (kni).* Time classification: cleavage cycle 11 (C11), cleavage cycle 12 (C12), cleavage cycle 13 (C13), cleavage cycle 14A (C14A); C14A is subdivided into eight equally spaced time classes (T1–T8). All time classes as defined in Wotton et al. (2014) for *M. abdita*, and Surkova et al. (2008) for *D. melanogaster*.

Boundary locations are indicated in % A–P position (where 0% is the anterior pole); differences in boundary position between species and domain widths are reported in % egg length (%EL).

**1. Boundary Naming Scheme**

To uniquely identify expression features, we enumerate each expression boundary for each gene starting from 0 at the most anterior boundary. The resulting naming scheme is shown in Figure 3. Measured positions of boundaries through time are reported in Section 3 below.

**
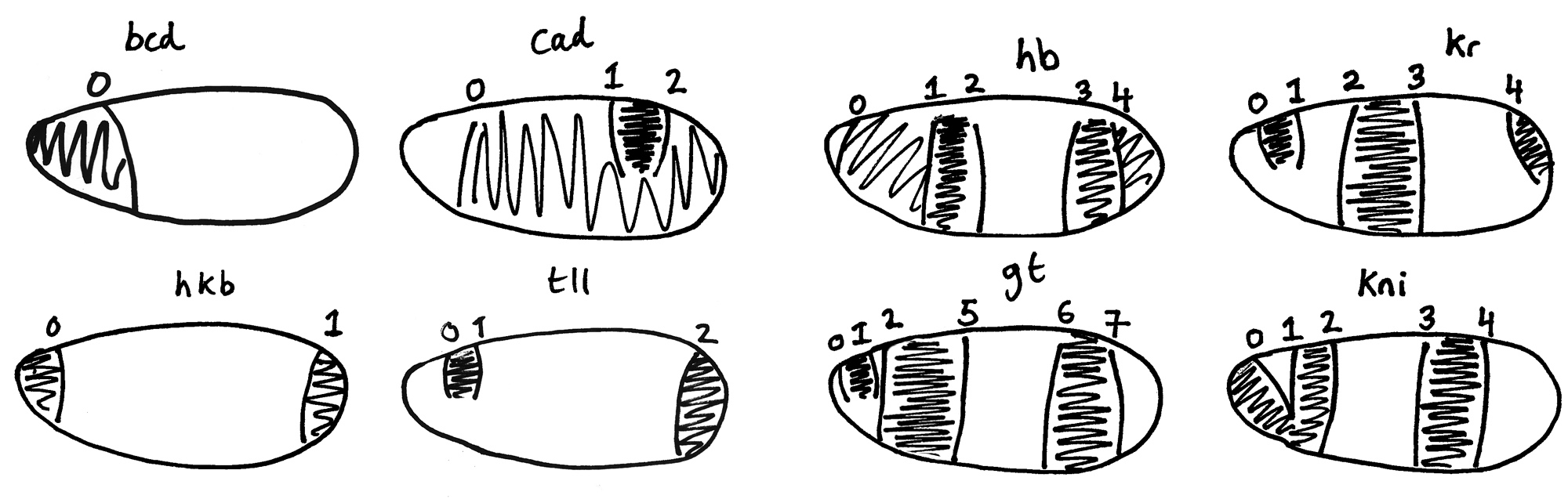
**

**Figure 1. Boundary naming scheme.** This figure displays schematic drawings of embryos showing expression boundary numbers and relative positions for each *M. abdita* gene listed in the tables in Section 3 of this document. *Gt* boundaries 3 and 4 are omitted to provide consistent numbering with homologous *D. melanogaster* boundaries (Crombach et al., 2012). Maternal inputs (*bcd, cad*) and terminal gap genes (*tll, hkb*) are shown on the left. Trunk gap genes (*hb, Kr, gt, kni*) are shown on the right. Embryos drawn anterior to the left, dorsal up.

**2. Boundary Position Tables**

These tables show positions of boundaries along the A–P axis (in %, where 0% is the anterior pole) for all expression boundaries in Figure 3. Boundary positions correspond to the position of inflection points for fitted splines (see Crombach et al., 2012a), for details). Our data cover cleavage cycles 11 (C11) to C14A. Time points are sorted into canonical time classes C11, C12, C13, and C14A-T1 to T8 as defined in Wotton et al. (2014). See Crombach et al. (2012) for equivalent *D. melanogaster* tables. na (not applicable): boundary not present during the respective time class. Dashes mean that no data is available for a given time class.

| **Gene: *bcd*** | |
| --- | --- |
| Time class | 0 |
| C11 | 23.008 |
| C12 | 15.628 |
| C13 | 12.469 |
| T1 | 11.961 |

| **Gene: *cad*** | | | |
| --- | --- | --- | --- |
| Time class | 0 | 1 | 2 |
| C11 | - | - | - |
| C12 | 33.806 | na | na |
| C13 | 31.146 | na | na |
| T1 | 31.644 | na | na |
| T2 | 30.369 | na | na |
| T3 | 31.004 | na | 90.707 |
| T4 | 31.396 | na | 89.844 |
| T5 | 31.300 | 79.230 | 89.553 |
| T6 | 32.170 | 80.345 | 89.113 |
| T7 | na | 82.338 | 89.741 |
| T8 | na | 79.530 | 86.720 |

| **Gene: *hkb*** | | |
| --- | --- | --- |
| Time class | 0 | 1 |
| C11 | - | - |
| C12 | 8.253 | 90.453 |
| C13 | 7.491 | 88.655 |
| T1 | 7.890 | 89.455 |
| T2 | 7.186 | 89.257 |
| T3 | 8.064 | 90.211 |
| T4 | - | - |
| T5 | 7.743 | 90.004 |
| T6 | 7.509 | 90.464 |
| T7 | 7.365 | 91.162 |
| T8 | 6.295 | 91.791 |

| **Gene: *tll*** | | | |
| --- | --- | --- | --- |
| Time class | 0 | 1 | 2 |
| C11 | - | - | - |
| C12 | na | na | 90.096 |
| C13 | na | na | 88.999 |
| T1 | na | na | 88.977 |
| T2 | na | na | 89.608 |
| T3 | na | na | 88.778 |
| T4 | na | na | 89.122 |
| T5 | na | na | 89.548 |
| T6 | 4.934 | 18.035 | na |
| T7 | 8.352 | 20.068 | na |
| T8 | na | 19.613 | na |

| **Gene: *hb*** | | | | | | |
| --- | --- | --- | --- | --- | --- | --- |
| Time class | 0 | 1 | 2 | 3 | 4 | 5 |
| C11 | na | na | 55.701 | na | na | na |
| C12 | na | na | 50.246 | 90.177 | na | na |
| C13 | 20.554 | na | 48.487 | 88.745 | na | na |
| T1 | 18.910 | na | 45.046 | 86.087 | na | na |
| T2 | 18.746 | na | 44.046 | 83.929 | na | na |
| T3 | 20.339 | na | 42.926 | 79.154 | na | na |
| T4 | 20.398 | na | 42.235 | 78.480 | na | na |
| T5 | 22.172 | na | 42.103 | 79.017 | na | na |
| T6 | 20.441 | na | 41.545 | 76.064 | 85.995 | na |
| T7 | 20.906 | na | 41.529 | 75.736 | 85.772 | na |
| T8 | 24.222 | na | 42.211 | 72.386 | na | 84.090 |

| **Gene: *Kr*** | | | | | |
| --- | --- | --- | --- | --- | --- |
| Time class | 0 | 1 | 2 | 3 | 4 |
| C11 | - | - | - | - | - |
| C12 | na | 9.669 | 39.551 | 62.586 | na |
| C13 | na | 8.766 | 40.162 | 63.498 | na |
| T1 | na | 13.527 | 40.227 | 61.074 | na |
| T2 | na | 12.801 | 39.851 | 60.550 | na |
| T3 | 6.312 | 15.000 | 38.597 | 58.004 | na |
| T4 | 8.417 | 15.983 | 38.751 | 57.984 | na |
| T5 | 10.392 | 16.333 | 38.296 | 54.691 | na |
| T6 | 11.736 | 17.118 | 37.727 | 51.543 | na |
| T7 | 11.562 | 17.379 | 37.285 | 51.332 | 91.880 |
| T8 | 12.214 | 18.047 | 36.961 | 50.659 | 88.318 |

| **Gene: *gt*** | | | | | | |
| --- | --- | --- | --- | --- | --- | --- |
| Time class | 0 | 1 | 2 | 5 | 6 | 7 |
| C11 | - | - | - | - | - | - |
| C12 | na | na | 14.031 | 33.727 | 73.341 | na |
| C13 | na | na | 15.312 | 34.523 | 72.036 | 88.722 |
| T1 | na | na | 13.551 | 32.488 | 69.693 | 86.169 |
| T2 | na | na | 14.693 | 34.287 | 70.638 | 86.046 |
| T3 | na | na | 14.440 | 33.141 | 69.914 | 85.801 |
| T4 | na | na | 16.584 | 34.927 | 68.294 | 84.040 |
| T5 | na | 7.413 | 16.872 | 31.915 | 64.993 | 78.532 |
| T6 | 4.503 | 8.006 | 23.953 | 33.527 | 64.425 | 74.844 |
| T7 | 3.506 | 9.265 | 23.990 | 32.417 | 63.242 | 72.760 |
| T8 | 5.788 | 10.058 | 25.619 | 33.248 | 62.895 | 71.026 |

| **Gene: *kni*** | | | | | |
| --- | --- | --- | --- | --- | --- |
| Time class | 0 | 1 | 2 | 3 | 4 |
| C11 | - | - | - | - | - |
| C12 | 7.769 | na | na | 60.608 | 77.555 |
| C13 | 8.742 | na | na | 58.531 | 74.536 |
| T1 | 7.100 | na | na | 57.371 | 71.037 |
| T2 | 8.155 | na | na | 56.256 | 71.315 |
| T3 | 8.609 | 23.770 | 26.784 | 57.623 | 70.128 |
| T4 | 8.343 | 22.102 | 26.896 | 55.817 | 67.810 |
| T5 | 8.855 | 22.654 | 26.869 | 54.695 | 66.393 |
| T6 | 9.050 | 22.913 | 28.217 | 54.173 | 65.891 |
| T7 | 8.607 | 24.211 | 28.356 | 53.358 | 64.404 |
| T8 | 9.057 | 23.359 | 28.386 | 53.592 | 63.523 |

**3. References**

Crombach, A., Wotton, K.R., Cicin-Sain, D., Ashyraliyev, M., Jaeger, J., 2012. Efficient reverse-engineering of a developmental gene regulatory network. PLoS Comput. Biol. 8, e1002589. doi:10.1371/journal.pcbi.1002589

Surkova, S., Kosman, D., Kozlov, K., Manu, Myasnikova, E., Samsonova, A. a, Spirov, A., Vanario-Alonso, C.E., Samsonova, M., Reinitz, J., 2008. Characterization of the *Drosophila* segment determination morphome. Dev. Biol. 313, 844–62. doi:10.1016/j.ydbio.2007.10.037

Wotton, K.R., Jiménez-Guri, E., García Matheu, B., Jaeger, J., 2014. A staging scheme for the development of the scuttle fly *Megaselia abdita*. PLoS One 9, e84421. doi:10.1371/journal.pone.0084421
