# Supplementary material for: Quantitative system drift compensates for altered maternal inputs to the gap gene network of the scuttle fly Megaselia abdita
Source: eLife. 2015 Jan 5;4:e04785. doi: 10.7554/eLife.04785 (PMC4337606; doi:10.7554/eLife.04785)
Supplement: Supplementary file 4. — RNAi dataset. DOI: http://dx.doi.org/10.7554/eLife.04785.021 [file elife04785s004.docx]

# Supplementary File 4:

# RNAi data set

This file contains plots of gap gene expression boundaries from RNAi-treated embryos, and tables indicating the position of these boundaries in relation to wild-type boundary positioning and variability.

In the tables numbers for each boundary, at each time class, are represented as (x, y, z) where x = boundaries more anterior than wild-type variability, y = boundaries more posterior than wild-type variability and z = total number of boundaries.

**Table of Contents**

1. *hunchback (hb)* RNAi knock-down

2. *giant (gt)* RNAi knock-down

3. *Krüppel (Kr)* RNAi knock-down

4. *knirps (kni)* RNAi knock-down

5. *tailless (tll)* RNAi knock-down

6. *huckebein (hkb)* RNAi knock-down

7. *tailless (tll):huckebein (hkb)* double RNAi knock-down

**1. *hunchback (hb)* RNAi knock-down**

**Figure 1: Expression plots—*hb* RNAi knock-down in *M. abdita*.** Graphs show extracted boundary positions for wild-type (grey), and *hb* RNAi-treated embryos (coloured) as in Figure 2C of the main text. See Table 2 (main text) for the number of embryos in the data set.

**Table 1: Boundary positions in *hb* RNAi-treated embryos compared to the range of positions in wild-type.** Columns represent distinct boundaries (numbered as in Supplementary File 3, Figure 3; rows represent time classes. Number triplets *(x, y, z)* indicate: *x* = boundaries more anterior, and *y* = boundaries more posterior than the wild-type range; *z =* total number of boundaries at each time class. Dashes: no data.

| *hb* RNAi:  *Kr* stain |  |  | anterior boundary of central domain | posterior boundary of central domain |
| --- | --- | --- | --- | --- |
|  | **0** | **1** | **2** | **3** |
| C11 | - | - | - | - |
| C12 | - | - | (1, 0, 2) | (0, 0, 2) |
| C13 | - | - | (0, 0, 4) | (4, 0, 4) |
| T1 | - | - | (5, 0, 5) | (2, 0, 5) |
| T2 | - | (0, 0, 1) | (1, 1, 9) | (3, 1, 9) |
| T3 | - | (1, 0, 1) | (6, 0, 7) | (3, 1, 7) |
| T4 | (0, 3, 4) | (0, 0, 5) | (3, 1, 9) | (3, 0, 9) |
| T5 | (0, 3, 7) | (0, 3, 7) | (3, 1, 11) | (8, 0, 11) |
| T6 | (0, 1, 4) | (0, 1, 4) | (1, 2, 4) | (1, 1, 4) |
| T7 | (0, 0, 1) | (0, 0, 1) | (1, 0, 1) | (0, 0, 1) |
| T8 | - | - | - | - |
| Total: | 16 | 19 | 52 | 52 |
| Anterior dis-placement: | - | 1/19 = 5% | 21/52 = 40% | 24/52 = 46% |
| Posterior dis-placement: | 7/16 = 43% | 4/19 = 21% | 5/52 = 10% | 3/52 = 6% |

**Table 1** (contd.)

| *hb* RNAi:  *gt* stain |  | anterior boundary of anterior domain | posterior boundary of anterior domain | anterior boundary of posterior domain | posterior boundary of posterior domain |
| --- | --- | --- | --- | --- | --- |
|  | **1** | **2** | **5** | **6** | **7** |
| C11 | - | - | - | - | - |
| C12 | - | - | - | - | - |
| C13 | - | (0, 2, 4) | (2, 1, 4) | (0, 3, 4) | - |
| T1 | - | (0, 0, 2) | (1, 0, 2) | (0, 0, 2) | - |
| T2 | - | - | - | - | - |
| T3 | - | (0, 0, 9) | (0, 3, 9) | (0, 3, 9) | (0, 0, 3) |
| T4 | - | (0, 3, 9) | (1, 0, 9) | (0, 6, 9) | - |
| T5 | (0, 1, 1) | (0, 0, 5) | (0, 5, 5) | (0, 5, 5) | (0, 2, 2) |
| T6 | - | (0, 0, 2) | (0, 0, 2) | (0, 2, 2) | (0, 1, 2) |
| T7 | - | (0, 0, 3) | (1, 1, 3) | (0, 0, 3) | (0, 0, 3) |
| T8 | (0, 0, 1) | (0, 0, 1) | (0, 0, 1) | (0, 0, 1) | (0, 1, 1) |
| Total | 2 | 35 | 35 | 35 | 11 of 35 |
| Anterior dis-placement: | - | - | 5/35 = 14% | - | - |
| Posterior dis-placement: | 1/2 = 50% | 5/35 = 14% | 10/35 = 29% | 19/35 = 54% | 4/35 = 11% |
| Fails to retract: | - | - | - | - | 24/35 = 69% |
| Total posterior boundary of posterior domain defects: |  |  |  |  | 28/35 = 80% |

| *hb* RNAi:  *kni* stain |  |  |  | anterior boundary of abdominal domain | posterior boundary of abdominal domain |
| --- | --- | --- | --- | --- | --- |
|  | **0** | **1** | **2** | **3** | **4** |
| C12 | - | - | - | - | - |
| C13 | (1, 1, 2) | - | - | (2, 0, 4) | (0, 1, 4) |
| T1 | (1, 0, 1) | - | - | (1, 0, 1) | (0, 0, 1) |
| T2 | (1, 0, 1) | - | - | (1, 0, 1) | (1, 0, 1) |
| T3 | (1, 0, 3) | - | - | (2, 0, 3) | (0, 0, 3) |
| T4 | (0, 0, 3) | - | - | (2, 0, 3) | (0, 0, 3) |
| T5 | (1, 0, 2) | (0, 1, 1) | (0, 0, 1) | (1, 0, 2) | (0, 1, 2) |
| T6 | - | - | - | - | - |
| T7 | - | - | - | - | - |
| T8 | - | - | - | - | - |
| Total: | 12 | 1 | 1 | 14 | 14 |
| Anterior dis-placement: | 5/12 = 42% | - | - | 9/14 = 64% | 1/14 = 7% |
| Posterior dis-placement: | 1/12 = 8% | 1/1 = 100% | - | - | 2/14 = 14% |

**2. *giant (gt)* RNAi knock-down**

**Figure 2: Expression plots—*gt* RNAi knock-down in *M. abdita*.** Graphs show extracted boundary positions for wild-type (grey), and *gt* RNAi-treated embryos (coloured) as in Figure 2C of the main text. See Table 2 (main text) for the number of embryos in the data set.

**Table 2: Boundary positions in *gt* RNAi-treated embryos compared to the range of positions in wild-type.** Columns represent distinct boundaries (numbered as in Supplementary File 3, Figure 3; rows represent time classes. Number triplets *(x, y, z)* indicate: *x* = boundaries more anterior, and *y* = boundaries more posterior than the wild-type range; *z =* total number of boundaries at each time class. Dashes: no data.

| *gt* RNAi:  *hb* stain | anterior boundary of anterior domain | posterior boundary of anterior domain | anterior boundary of posterior domain | posterior boundary of posterior domain |
| --- | --- | --- | --- | --- |
|  | **0** | **2** | **3** | **4** |
| C11 | - | - | - | - |
| C12 | - | (2, 0, 3) | (1, 0, 1) | - |
| C13 | - | (0, 0, 2) | - | - |
| T1 | - | - | - | - |
| T2 | - | - | - | - |
| T3 | (1, 0, 9) | (0, 0, 11) | (0, 7, 11) | - |
| T4 | (1, 0, 2) | (0, 1, 6) | (0, 5, 5) | - |
| T5 | (4, 0, 4) | (1, 1, 5) | (2, 3, 5) | - |
| T6 | - | (1, 0, 2) | (0, 1, 2) | - |
| T7 | - | (0, 0, 1) | (0, 1, 1) | (0, 1, 1) |
| T8 | - | - | - | - |
| Total | 15 | 30 | 28 | 1 |
| Anterior dis-placement: | 6/15 = 40% | 4/30 = 13% | 3/28 = 11% | - |
| Posterior dis-placement: | - | 2/30 = 7% | 17/28 = 60% | 1/1 = 100% |

| *gt* RNAi:  *Kr* stain |  |  | anterior boundary of central domain | posterior boundary of central domain |
| --- | --- | --- | --- | --- |
|  | **0** | **1** | **2** | **3** |
| C11 | - | - | - | - |
| C12 | - | - | - | - |
| C13 | - | - | - | - |
| T1 | - | - | - | - |
| T2 | - | - | (0, 2, 4) | (2, 0, 4) |
| T3 | - | - | (0, 1, 2) | (1, 0, 2) |
| T4 | (0, 1, 1) | (0, 0, 1) | (0, 3, 4) | (0, 0, 4) |
| T5 | (1, 0, 2) | (0, 0, 2) | (0, 1, 3) | (1, 0, 3) |
| T6 | (0, 0, 1) | (0, 0, 1) | (0, 0, 1) | (0, 0, 1) |
| T7 | (0, 0, 3) | (0, 0, 3) | (0, 0, 3) | (1, 0, 3) |
| T8 | - | - | - | - |
| Total | 7 | 7 | 17 | 17 |
| Anterior dis-placement: | 1/7 = 14% | - | - | 5/17 = 29% |
| Posterior dis-placement: | 1/7 = 14% | - | 7/17 = 41% | - |

**Table 2** (contd.)

| *gt* RNAi:  *kni* stain |  |  |  | anterior boundary of abdominal domain | posterior boundary of abdominal domain |
| --- | --- | --- | --- | --- | --- |
|  | **0** | **1** | **2** | **3** | **4** |
| C11 | - | - | - | - | - |
| C12 | - | - | - | - | - |
| C13 | - | - | - | (0, 0, 1) | (0, 0, 1) |
| T1 | (0, 0, 1) | - | - | (0, 1, 2) | (0, 0, 2) |
| T2 | (0, 0, 1) | - | - | (0, 0, 2) | (0, 1, 2) |
| T3 | (0, 0, 4) | - | - | (0, 0, 5) | (0, 0, 5) |
| T4 | (0, 0, 2) | - | - | (0, 0, 4) | (0, 2, 4) |
| T5 | (0, 0, 3) | (0, 1, 2) | (0, 0, 2) | (0, 1, 6) | (0, 3, 6) |
| T6 | (0, 0, 2) | (0, 1, 1) | (0, 0, 1) | (0, 1, 3) | (0, 3, 3) |
| T7 | (0, 0, 1) | (0, 0, 1) | (0, 0, 1) | (0, 0, 1) | (0, 1, 1) |
| T8 | - | (0, 0, 1) | (1, 0, 1) | (1, 0, 1) | (0, 1, 1) |
| Total | 14 | 5 | 5 | 25 | 25 |
| Anterior dis-placement: | - | - | 1/5 = 20% | 3/25 = 12% | - |
| Posterior dis-placement: | - | 2/5 = 40% | - | - | 11/25 = 44% |

**3. *Krüppel (Kr)* RNAi knock-down**

**Figure 3: Expression plots—*Kr* RNAi knock-down in *M. abdita*.** Graphs show extracted boundary positions for wild-type (grey), and *Kr* RNAi-treated embryos (coloured) as in Figure 2C of the main text. See Table 2 (main text) for the number of embryos in the data set.

**Table 3: Boundary positions in *Kr* RNAi-treated embryos compared to the range of positions in wild-type.** Columns represent distinct boundaries (numbered as in Supplementary File 3, Figure 3; rows represent time classes. Number triplets *(x, y, z)* indicate: *x* = boundaries more anterior, and *y* = boundaries more posterior than the wild-type range; *z =* total number of boundaries at each time class. Dashes: no data.

| *Kr* RNAi:  *hb* stain | anterior boundary of anterior domain | posterior boundary of anterior domain | anterior boundary of posterior domain | posterior boundary of posterior domain |
| --- | --- | --- | --- | --- |
|  | **0** | **2** | **3** | **4** |
| C11 | - | - | - | - |
| C12 | - | - | - | - |
| C13 | - | (0, 1, 3) | (1, 0, 1) | - |
| T1 | (0, 1, 2) | (0, 0, 3) | (0, 1, 2) | - |
| T2 | (0, 2, 2) | (0, 2, 6) | (0, 0, 6) | - |
| T3 | (0, 1, 6) | (0, 6, 13) | (0, 3, 13) | - |
| T4 | (0, 2, 5) | (0, 8, 9) | (3, 5, 9) | - |
| T5 | (4, 0, 5) | (0, 4, 7) | (1, 2, 7) | - |
| T6 | - | - | - | - |
| T7 | - | - | - | - |
| T8 | - | - | - | - |
| Total: | 20 | 41 | 38 | - |
| Anterior dis-placement: | 6/20 = 30% | - | 5/38 = 13% | - |
| Posterior dis-placement: | 4/20 = 20% | 21/41 = 51% | 11/38 = 29% | - |

**Table 3** (contd.)

| *Kr* RNAi:  *gt* stain | anterior boundary of anterior domain | posterior boundary of anterior domain | | anterior boundary of posterior domain | posterior boundary of posterior domain |
| --- | --- | --- | --- | --- | --- |
|  | **2** | **5** | **6** | | **7** |
| C11 | - | - | - | | - |
| C12 | - | - | - | | - |
| C13 | - | (0, 1, 1) | (1, 0, 1) | | - |
| T1 | - | - | - | | - |
| T2 | (0, 0, 3) | (0, 3, 3) | (1, 0, 3) | | (1, 0, 2) |
| T3 | (0, 0, 5) | (0, 5, 6) | (4, 0, 6) | | (1, 0, 2) |
| T4 | (0, 3, 8) | (0, 6, 8) | (2, 1, 8) | | (0, 0, 5) |
| T5 | (0, 0, 2) | (0, 2, 2) | (0, 1, 2) | | (0, 2, 2) |
| T6 | - | - | - | | - |
| T7 | - | - | - | | - |
| T8 | - | - | - | | - |
| Total: | 18 | 20 | 20 | | 11 of 20 |
| Anterior dis-placement | - | - | 8/20 = 40% | | 2/20 = 10% |
| Posterior dis-placement | 3/18 = 17% | 17/20 = 85% | 2/20 = 10% | | 2/20 = 10% |
| Fails to retract: | - | - | - | | 9/20 = 45% |
| Total posterior boundary of posterior domain defects: |  |  |  | | 11/20 = 55%  or 13/20 = 65% including anterior displacements |

| *Kr* RNAi:  *kni* stain |  |  |  | anterior boundary of abdominal domain | posterior boundary of abdominal domain |
| --- | --- | --- | --- | --- | --- |
|  | **0** | **1** | **2** | **3** | **4** |
| C11 | - | - | - | - | - |
| C12 | - | - | - | - | - |
| C13 | (0, 0, 1) | - | - | (0, 0, 4) | (1, 0, 4) |
| T1 | - | - | - | (0, 0, 1) | (0, 0, 1) |
| T2 | (1, 0, 4) | - | - | (0, 0, 6) | (3, 0, 6) |
| T3 | (0, 0, 6) | - | - | (0, 0, 6) | (0, 0, 6) |
| T4 | (0, 0, 3) | - | - | (0, 1, 3) | (0, 0, 3) |
| T5 | (1, 0, 1) | (0, 1, 1) | (0, 0, 1) | (0, 0, 1) | (1, 0, 1) |
| T6 | - | - | - | - | - |
| T7 | - | - | - | - | - |
| T8 | - | - | - | - | - |
| Total: | 15 | 1 | 1 | 21 | 21 |
| Anterior dis-placement | 1/15 = 7% | - | - | - | 3/21 = 14% |
| Posterior dis-placement | - | 1/1 = 100% | - | 1/21 = 5% | - |

**4. *knirps (kni)* RNAi knock-down**

**Figure 4: Expression plots—*kni* RNAi knock-down in *M. abdita*.** Graphs show extracted boundary positions for wild-type (grey), and *kni* RNAi-treated embryos (coloured) as in Figure 2C of the main text. See Table 2 (main text) for the number of embryos in the data set.

**Table 4: Boundary positions in *kni* RNAi-treated embryos compared to the range of positions in wild-type.** Columns represent distinct boundaries (numbered as in Supplementary File 3, Figure 3; rows represent time classes. Number triplets (x, y, z) indicate: x = boundaries more anterior, and y = boundaries more posterior than the wild-type range; z = total number of boundaries at each time class. Dashes: no data.

| *kni* RNAi:  *hb* stain | anterior boundary of anterior domain | posterior boundary of anterior domain | anterior boundary of posterior domain | posterior boundary of posterior domain |
| --- | --- | --- | --- | --- |
|  | **0** | **2** | **3** | **4** |
| C11 | - | - | - | - |
| C12 | - | - | - | - |
| C13 | - | (0, 1, 2) | (1, 0, 1) | - |
| T1 | - | (0, 1, 1) | - | - |
| T2 | (0, 0, 1) | (0, 0, 3) | (2, 0, 3) | - |
| T3 | (0, 0, 10) | (0, 2, 10) | (0, 0, 10) | - |
| T4 | (1, 4, 9) | (0, 7, 10) | (8, 0, 10) | - |
| T5 | (12, 2, 14) | (0, 9, 14) | (13, 1, 14) | - |
| T6 | (1, 3, 10) | (0, 8, 10) | (7, 1, 10) |  |
| T7 | (1, 1, 4) | (0, 2, 4) | (4, 0, 4) | - |
| T8 | - | - | - | - |
| Total: | 48 | 54 | 52 | - |
| Anterior dis-placement: | 15/48 = 31% | - | 35/52 = 67% | - |
| Posterior dis-placement: | 10/48 = 21% | 30/54 = 56% | 2/52 = 4% | - |

**Table 4** (contd.)

| *kni* RNAi:  *gt* stain |  | | anterior boundary of anterior domain | posterior boundary of anterior domain | anterior boundary of posterior domain | posterior boundary of posterior domain |
| --- | --- | --- | --- | --- | --- | --- |
|  | **1** | **2** | | **5** | **6** | **7** |
| C11 | - | - | | - | - | - |
| C12 | - | - | | - | - | - |
| C13 | - | - | | - | - | - |
| T1 | - | (0, 1, 1) | | (0, 1, 1) | (0, 1, 1) | - |
| T2 | - | (0, 0, 4) | | (0, 2, 4) | (0,2, 4) | (1, 0, 1) |
| T3 | - | - | | - | - | - |
| T4 | - | (0, 2, 8) | | (0, 2, 9) | (0, 8, 9) | (0, 0, 1) |
| T5 | (0, 1, 3) | (0, 0, 8) | | (0, 3, 8) | (0, 5, 8) | (0, 2, 5) |
| T6 | (0, 0, 7) | (0, 0, 8) | | (0, 2, 8) | (0, 6, 6) | (0, 4, 5) |
| T7 | (0, 0, 1) | (1, 0, 2) | | (0, 2, 2) | (0, 2, 2) | - |
| T8 | - | - | | - | - | - |
| Total: | 11 | 31 | | 32 | 30 | 12 of 30 |
| Anterior dis-placement | - | 1/31 = 3% | | - | - | 1/30 = 3% |
| Posterior dis-placement | 1/11 = 9% | 3/31 = 10% | | 12/32 = 34% | 24/30 = 80% | 6/30 = 20% |
| Fails to retract: | - | - | | - | - | 18/30= 60% |
| Total posterior boundary of posterior domain defects: |  |  | |  |  | 24/30 = 80% |

| *kni* RNAi:  *Kr* stain |  |  | anterior boundary of central domain | posterior boundary of central domain |
| --- | --- | --- | --- | --- |
|  | **0** | **1** | **2** | **3** |
| C11 | - | - | - | - |
| C12 | - | - | - | - |
| C13 | - | - | (0, 1, 1) | (0, 1, 1) |
| T1 | - | - | - | - |
| T2 | - | - | - | - |
| T3 | - | - | (0, 7, 9) | (0, 8, 9) |
| T4 | (0, 4, 4) | (0, 0, 4) | (0, 5, 16) | (0, 6, 16) |
| T5 | (0, 1, 4) | (0, 1, 4) | (0, 2, 6) | (0, 3, 6) |
| T6 | (0, 1, 3) | (0, 2, 3) | (1, 4, 5) | (0, 5, 5) |
| T7 | (0, 0, 3) | (0, 0, 3) | (0, 1, 3) | (0, 3, 3) |
| T8 | - | - | - | - |
| Total: | 14 | 14 | 40 | 40 |
| Anterior dis-placement | - | - | 1/40 = 3% | - |
| Posterior dis-placement: | 6/14 = 43% | 3/14 = 21% | 20/40 = 50% | 26/40 = 65% |

**5. *tailless (tll)* RNAi knock-down**

**Figure 5: Expression plots—*tll* RNAi knock-down in *M. abdita*.** Graphs show extracted boundary positions for wild-type (grey), and *tll* RNAi-treated embryos (coloured) as in Figure 2C of the main text. See Table 2 (main text) for the number of embryos in the data set.

**Table 5: Boundary positions in *tll* RNAi-treated embryos compared to the range of positions in wild-type.** Columns represent distinct boundaries (numbered as in Supplementary File 3, Figure 3; rows represent time classes. Number triplets *(x, y, z)* indicate: *x* = boundaries more anterior, and *y* = boundaries more posterior than the wild-type range; *z =* total number of boundaries at each time class. Dashes: no data.

| *tll* RNAi:  *hb* stain | anterior boundary of anterior domain | posterior boundary of anterior domain | anterior boundary of posterior domain | posterior boundary of posterior domain |
| --- | --- | --- | --- | --- |
|  | **0** | **2** | **3** | **4** |
| C11 | - | - | - | - |
| C12 | - | - | - | - |
| C13 | (0, 1, 1) | (0, 0, 1) | (1, 0, 1) | - |
| T1 | (0, 1, 4) | (0, 0, 4) | (0, 0, 2) | - |
| T2 | (1, 1, 4) | (0, 0, 5) | (0, 0, 4) | - |
| T3 | (1, 0, 11) | (0, 1, 11) | (0, 3, 10) | - |
| T4 | (0, 3, 8) | (1, 4, 8) | (1, 7, 8) | - |
| T5 | (2, 1, 3) | (0, 0, 3) | (0, 1, 3) | - |
| T6 | - | - | - | - |
| T7 | (0, 0, 1) | (0, 1, 1) | (0, 1, 1) | - |
| T8 | - | - | - | - |
| Total | 32 | 33 | 29 | - |
| Anterior dis-placement: | 4/32 = 13% | 1/33 = 3% | 2/29 = 7% | - |
| Posterior dis-placement: | 7/32 = 22% | 6/33 = 18% | 12/29 = 41% | - |
| Posterior domain absent: | - | - | 4/28 = 14% | - |
| Fails to retract/clear: | - | - | - | 1/1 = 100%* |

* 1 embryo at T7 shows no clearing of *hb* expression from the pole

| *tll* RNAi:  *Kr* stain | |  |  | anterior boundary of central domain | posterior boundary of central domain |
| --- | --- | --- | --- | --- | --- |
|  | **0** | | **1** | **2** | **3** |
| C11 | - | | - | - | - |
| C12 | - | | - | - | - |
| C13 | - | | - | (0, 1, 1) | (0, 0, 1) |
| T1 | - | | - | (0, 0, 1) | (0, 0, 1) |
| T2 | - | | - | (0, 1, 2) | (1, 0, 2) |
| T3 | - | | - | (1, 3, 9) | (4, 1, 9) |
| T4 | - | | - | (0, 2, 2) | (0, 0, 2) |
| T5 | - | | - | (0, 0, 2) | (1, 0, 2) |
| T6 | - | | - | - | - |
| T7 | (0, 0, 1) | | (0, 0, 1) | (0, 0, 1) | (1, 0, 1) |
| T8 | - | | - | - | - |
| Total: | 1 | | 1 | 18 | 18 |
| Anterior dis-placement: | - | | - | 1/18 = 6% | 6/18 = 33% |
| Posterior dis-placement: | - | | - | 7/18 = 38% | 1/18 = 6% |

**Table 5** (contd.)

| *tll* RNAi:  *gt* stain |  | anterior boundary of anterior domain | posterior boundary of anterior domain | anterior boundary of posterior domain | posterior boundary of posterior domain |
| --- | --- | --- | --- | --- | --- |
|  | **1** | **2** | **5** | **6** | **7** |
| C11 | - | - | - | - | - |
| C12 | - | - | - | - | - |
| C13 | - | - | - | - | - |
| T1 | - | (0, 2, 2) | (0, 0, 2) | (0, 0, 2) | - |
| T2 | - | (0, 4, 8) | (2, 1, 8) | (0, 1, 8) | (0, 0, 1) |
| T3 | - | (0, 0, 6) | (4, 0, 6) | (0, 2, 6) | (0, 1, 3) |
| T4 | - | (0, 0, 5) | (0, 0, 5) | (0, 5, 5) | (0, 1, 2) |
| T5 | (0, 0, 2) | (0, 0, 7) | (1, 3, 7) | (0, 4, 7) | (0, 6, 7) |
| T6 | (1, 0, 1) | (0, 0, 2) | (1, 0, 2) | (1, 1, 2) | (0, 1, 2) |
| T7 | - | - | - | - | - |
| T8 | - | - | - | - | - |
| Total | 3 | 30 | 30 | 30 | 15 of 30 |
| Anterior dis-placement: | 1/3 = 33% | - | 8/30 = 27% | 1/30 = 3% | - |
| Posterior dis-placement: | - | 6/30 = 20% | 4/30 = 13% | 13/30 = 43% | 9/30 = 30% |
| Fails to retract: | - | - | - | - | 15/30 = 50% |
| Total posterior boundary of posterior domain defects: | - | - | - | - | 24/30 = 80% |

| *tll* RNAi:  *kni* stain |  |  |  | anterior boundary of abdominal domain | posterior boundary of abdominal domain |
| --- | --- | --- | --- | --- | --- |
|  | **0** | **1** | **2** | **3** | **4** |
| C11 | - | - | - | - | - |
| C12 | - | - | - | - | - |
| C13 | - | - | - | (0, 1, 2) | (0, 0, 2) |
| T1 | - | - | - | - | - |
| T2 | (1, 0, 4) | - | - | (0, 0, 6) | (2, 1, 6) |
| T3 | (0, 0, 11) | - | - | (0, 0, 21) | (2, 0, 21) |
| T4 | (3, 0, 8) | - | - | (0, 3, 9) | (0, 1, 9) |
| T5 | (1, 0, 1) | - | - | (0, 0, 2) | (0, 0, 2) |
| T6 | (1, 0, 2) | (0, 1, 1) | (0, 0, 1) | (0, 2, 3) | (0, 1, 3) |
| T7 | (0, 0, 1) | (1, 0, 1) | (1, 0, 1) | (0, 0, 1) | (0, 0, 1) |
| T8 | - | - | - | - | - |
| Total: | 27 | 2 | 2 | 44 | 44 |
| Anterior dis-placement: | 6/27 = 22% | 1/2 = 50% | 1/2 = 50% | - | 4/44 = 9% |
| Posterior dis-placement: | - | 1/2 = 50% | - | 6/44 = 14% | 3/44 = 7% |

**6. *huckebein (hkb)* RNAi knock-down**

**Figure 6: Expression plots—*hkb* RNAi knock-down in *M. abdita*.** Graphs show extracted boundary positions for wild-type (grey), and *hkb* RNAi-treated embryos (coloured) as in Figure 2C of the main text. See Table 2 (main text) for the number of embryos in the data set.

**Table 6: Boundary positions in *hkb* RNAi-treated embryos compared to the range of positions in wild-type.** Columns represent distinct boundaries (numbered as in Supplementary File 3, Figure 3; rows represent time classes. Number triplets (x, y, z) indicate: x = boundaries more anterior, and y = boundaries more posterior than the wild-type range; z = total number of boundaries at each time class. Dashes: no data.

| *hkb* RNAi:  *hb* stain | anterior boundary of anterior domain | posterior boundary of anterior domain | anterior boundary of posterior domain | posterior boundary of posterior domain |
| --- | --- | --- | --- | --- |
|  | **0** | **2** | **3** | **4** |
| C11 | - | - | - | - |
| C12 | - | (1, 0, 1) | - | - |
| C13 | - | (0, 0, 1) | - | - |
| T1 | - | (0, 0, 2) | (0, 1, 1) | - |
| T2 | (1, 0, 1) | (0, 0, 2) | (0, 2, 2) | - |
| T3 | (0, 0, 4) | (2, 0, 4) | (0, 3, 4) | - |
| T4 | (1, 0, 3) | (0, 1, 3) | (0, 3, 3) | - |
| T5 | (1, 0, 1) | (0, 0, 1) | (0, 1, 1) | - |
| T6 | - | (0, 0, 1) | (0, 1, 1) | - |
| T7 | (1, 1, 4) | (2, 0, 4) | (0, 4, 4) | - |
| T8 | (3, 3, 6) | (1, 1, 6) | (0, 6, 6) | (0, 2, 2) |
| Total | 19 | 25 | 22 | 2 of 11 (T6-T8*) |
| Anterior dis-placement: | 7/19 = 37% | 6/25 = 24% | - | - |
| Posterior dis-placement: | 4/19 = 21% | 2/25 = 8% | 21/22 = 95% | 2/11 = 18% |
| Fail to retract/clear: |  |  |  | 9/11 = 81% |
| Total posterior boundary of posterior domain defects: |  |  |  | 11/11 = 100% |

* During T6-T8 *hb* expression clears from the posterior pole

| *hkb* RNAi:  *Kr* stain | anterior boundary of central domain | posterior boundary of central domain |
| --- | --- | --- |
|  | 2 | 3 |
| C11 | - | - |
| C12 | - | - |
| C13 | - | - |
| T1 | - | - |
| T2 | (0, 2, 2) | (2, 0, 2) |
| T3 | (0, 4, 4) | (2, 0, 4) |
| T4 | (0, 1, 1) | (0, 0, 1) |
| T5 | (0, 1, 2) | (2, 0, 2) |
| T6 | (0, 1, 1) | (0, 0, 1) |
| T7 | - | - |
| T8 | - | - |
| Total: | 10 | 10 |
| Anterior displacement: | - | 6/10 = 60% |
| Posterior displacement: | 10/10 = 100% | - |

**Table 6** (contd.)

| *hkb* RNAi:  *gt* stain | anterior boundary of anterior domain | posterior boundary of anterior domain | anterior boundary of posterior domain | posterior boundary of posterior domain |
| --- | --- | --- | --- | --- |
|  | **2** | **5** | **6** | **7** |
| C11 | - | - | - | - |
| C12 | - | - | - | - |
| C13 | - | - | - | - |
| T1 | (0, 2, 2) | (0, 1, 2) | (0, 0, 2) | - |
| T2 | (0, 1, 1) | (0, 1, 1) | (0, 1, 1) | - |
| T3 | (0, 0, 6) | (3, 0, 6) | (0, 6, 6) | - |
| T4 | (0, 1, 1) | (0, 0, 1) | (0, 1, 1) | - |
| T5 | - | - | - | - |
| T6 | - | - | - | - |
| T7 | - | - | - | - |
| T8 | - | - | - | - |
| Total: | 10 | 10 | 10 | 0 of10 |
| Anterior dis-placement: | - | 3/10 = 30% | - | - |
| Posterior dis-placement | 4/10 = 40% | - | 8/10 = 80% | - |
| Fails to retract: | - | - | - | 10/10 = 100% |

| *hkb* RNAi:  *kni* stain |  | anterior boundary of abdominal domain | posterior boundary of abdominal domain |
| --- | --- | --- | --- |
|  | **0** | **3** | **4** |
| C11 | - | - | - |
| C12 | - | - | - |
| C13 | - | - | - |
| T1 | - | - | - |
| T2 | - | (0, 0, 1) | (0, 0, 1) |
| T3 | (2, 0, 2) | (0, 0, 4) | (0, 0, 4) |
| T4 | (1, 0, 1) | (0, 1, 2) | (0, 0, 2) |
| T5 | - | (0, 2, 2) | (0, 0, 2) |
| T6 | - | (0, 1, 1) | (0, 0, 1) |
| T7 | - | - | - |
| T8 | - | - | - |
| Total: | 3 | 10 | 10 |
| Anterior dis-placement: | 3/3 = 100% | - | - |
| Posterior dis-placement: | - | 4/10 = 40% | - |

**7. *tailless (tll):huckebein (hkb)* double RNAi knock-down**

**Figure 7: Expression plots—*tll:hkb* double RNAi knock-down in *M. abdita*.** Graphs show extracted boundary positions for wild-type (grey), and *tll:hkb* RNAi-treated embryos (coloured) as in Figure 2C of the main text. See Table 2 (main text) for the number of embryos in the data set.

**Table 7: Boundary positions in *tll:hkb* RNAi-treated embryos compared to the range of positions in wild-type.** Columns represent distinct boundaries (numbered as in Supplementary File 3, Figure 3; rows represent time classes. Number triplets (x, y, z) indicate: x = boundaries more anterior, and y = boundaries more posterior than the wild-type range; z = total number of boundaries at each time class. Dashes: no data.

| *tll:hkb* RNAi:  *hb* stain | anterior boundary of anterior domain | posterior boundary of anterior domain | anterior boundary of posterior domain | posterior boundary of posterior domain |
| --- | --- | --- | --- | --- |
|  | **0** | **2** | **3** | **4** |
| C11 | - | - | - | - |
| C12 | - | (0, 0, 1) | - | - |
| C13 | - | - | - | - |
| T1 | - | - | - | - |
| T2 | - | (0, 1, 2) | (0, 1, 1) | - |
| T3 | (0, 0, 1) | (0, 0, 1) | (0, 1, 1) | - |
| T4 | (0, 0, 1) | (0, 3, 6) | (0, 6, 6) | - |
| T5 | (1, 0, 1) | (0, 1, 1) | (0, 1, 1) | - |
| T6 | (0, 0, 1) | (0, 1, 1) | (0, 1, 1) | - |
| T7 | - | (0, 1, 2) | (0, 2, 2) | - |
| T8 | (3, 0, 3) | (0, 0, 3) | (0, 3, 3) | (0, 3, 3) |
| Total | 7 | 17 | 15 | 3 of 6 (T6-T8) |
| Anterior dis-placement: | 4/7 = 57% | - | - | - |
| Posterior dis-placement: | - | 7/17 = 41% | 15/15 = 100% | 3/6 = 50% |
| Fail to retract/clear: | - | - | - | 3/6 = 50% |
| Total posterior boundary of posterior domain defects: | - | - | - | 6/6 = 100% |

* During T6-T8 *hb* expression clears from the posterior pole

**Table 7** (contd.)

| *tll:hkb* RNAi:  *Kr* stain |  |  | anterior boundary of central domain | posterior boundary of central domain |
| --- | --- | --- | --- | --- |
|  | **0** | **1** | **2** | **3** |
| C11 | - | - | - | - |
| C12 | - | - | - | - |
| C13 | - | - | (0, 0, 1) | (1, 0, 1) |
| T1 | - | - | (0, 0, 1) | (0, 0, 1) |
| T2 | - | (2, 0, 4) | (0, 4, 7) | (0, 2, 7) |
| T3 | (0, 1, 2) | (4, 0, 5) | (0, 4, 6) | (0, 2, 6) |
| T4 | 0, 0, 3) | (1, 0, 4) | (0, 3, 4) | (0, 0, 4) |
| T5 | (0, 0, 2) | (0, 0, 2) | (0, 1, 2) | (1, 0, 2) |
| T6 | - | - | - | - |
| T7 | - | - | - | - |
| T8 | - | - | - | - |
| Total: | 7 | 15 | 21 | 21 |
| Anterior dis-placement: | - | 7/15 = 47% | - |  |
| Posterior dis-placement: | 1/7 = 14% | - | 12/21 = 57% | 4/21 = 19% |

| *tll:hkb* RNAi:  *gt* stain | anterior boundary of anterior domain | posterior boundary of anterior domain | anterior boundary of posterior domain | posterior boundary of posterior domain |
| --- | --- | --- | --- | --- |
|  | **2** | **5** | **6** | **7** |
| C11 | - | - | - | - |
| C12 | - | - | - | - |
| C13 | (0, 1, 1) | (0, 0, 4) | (0, 4, 4) | - |
| T1 | (0, 1, 1) | (0, 1, 1) | (0, 0, 1) | - |
| T2 | (0, 0, 3) | (0, 1, 3) | (0, 2, 3) | - |
| T3 | (0, 0, 12) | (1, 0, 12) | (0, 3, 12) | (0, 0, 1) |
| T4 | (0, 1, 9) | (0, 0, 9) | (0, 5, 9) | - |
| T5 | (0, 0, 5) | (0, 1, 5) | (0, 5, 5) | - |
| T6 | (0, 0, 1) | (0, 0, 1) | (0, 1, 1) | (0, 1, 1) |
| T7 | - | - | - | - |
| T8 | (1, 0, 1) | (0, 0, 1) | (0, 1, 1) | (0, 1, 1) |
| Total: | 33 | 36 | 36 | 3 of 36 |
| Anterior dis-placement: | 1/33 = 3% | 1/36 = 3% | - | - |
| Posterior dis-placement: | 3/33 = 9% | 3/36 = 8% | 21/36 = 58% | 2/36 = 6% |
| Fails to retract: | - | - | - | 33/36 = 91% |
| Total posterior boundary of posterior domain defects: | - | - | - | 35/36 = 97% |

**Table 7** (contd.)

| *tll:hkb* RNAi:  *kni* stain |  |  |  | anterior boundary of abdominal domain | posterior boundary of abdominal domain |
| --- | --- | --- | --- | --- | --- |
|  | **0** | **1** | **2** | **3** | **4** |
| C11 | - | - | - | - | - |
| C12 | - | - | - | - | - |
| C13 | - | - | - | - | - |
| T1 | - | - | - | - | - |
| T2 | - | - | - | - | - |
| T3 | (0, 0, 4) | - | - | (0, 2, 4) | (0, 2, 4) |
| T4 | (2, 0, 7) | (0, 3, 3) | (1, 1, 3) | (0, 3, 8) | (0, 2, 8) |
| T5 | (1, 0, 2) | (0, 1, 2) | (0, 1, 2) | (0, 2, 2) | (0, 2, 2) |
| T6 | (0, 0, 1) | (0, 0, 1) | (0, 0, 1) | (0, 2, 2) | (0, 2, 2) |
| T7 | - | - | - | - | - |
| T8 | - | - | - | - | - |
| Total: | 14 | 6 | 6 | 16 | 16 |
| Anterior dis-placement: | 3/14 = 21% | - | 1/6 = 17% | - | - |
| Posterior dis-placement | - | 4/6 = 67% | 2/6 = 33% | 16/16 =100% | 16/16 =100% |
